# Genome Sequence of Bacteriophage Adumb2043, Isolated from Arthrobacter globiformis in Southern Colorado

**DOI:** 10.1128/MRA.00776-21

**Published:** 2021-10-14

**Authors:** Darien Brown, Shannon Isenhart, Auremie Kleven, Adam Gillison, Lee Anne Martínez, Amaya García Costas

**Affiliations:** a Department of Biology, Colorado State University-Pueblo, Pueblo, Colorado, USA; DOE Joint Genome Institute

## Abstract

We report the discovery and genome sequence of phage Adumb2043, a siphovirus infecting Arthrobacter globiformis, B2979-SEA. Adumb2043 was isolated from soil collected in Colorado Springs, Colorado. The genome has a length of 43,100 bp and contains 68 predicted protein-coding genes and no tRNA genes. Adumb2043 is related to actinobacteriophages Elezi and London.

## ANNOUNCEMENT

The phylum *Actinobacteria* comprises a morphologically, phylogenetically, and physiologically diverse group of Gram-positive bacteria that are mostly found in terrestrial, aquatic, and animal microbiomes (1). Many *Actinobacteria* strains are of human interest due to their medical, agricultural, or biotechnological applications or roles in disease (1–3). Like other bacteria, actinobacteria are susceptible to phage infection, which likely affects their ecology, evolution, and life cycle (4). The Science Education Alliance-Phage Hunters Advancing Genomics and Evolutionary Science (SEA-PHAGES) program spearheads a phage discovery and genome-sequencing project for these actinophages, with nearly 4,000 genomes having been sequenced to date (5, 6) (https://phagesdb.org). Most of these actinophages infect Mycobacterium smegmatis mc^2^155, the model organism for studying Mycobacterium tuberculosis. Here, we present the genome sequence of an actinophage infecting the soil actinobacterium Arthrobacter globiformis B2979-SEA, from the order *Micrococcales* (7). Expanding the diversity of sequenced actinophages provides data needed to fully understand the evolution and ecology of these important phages.

**FIG 1:**
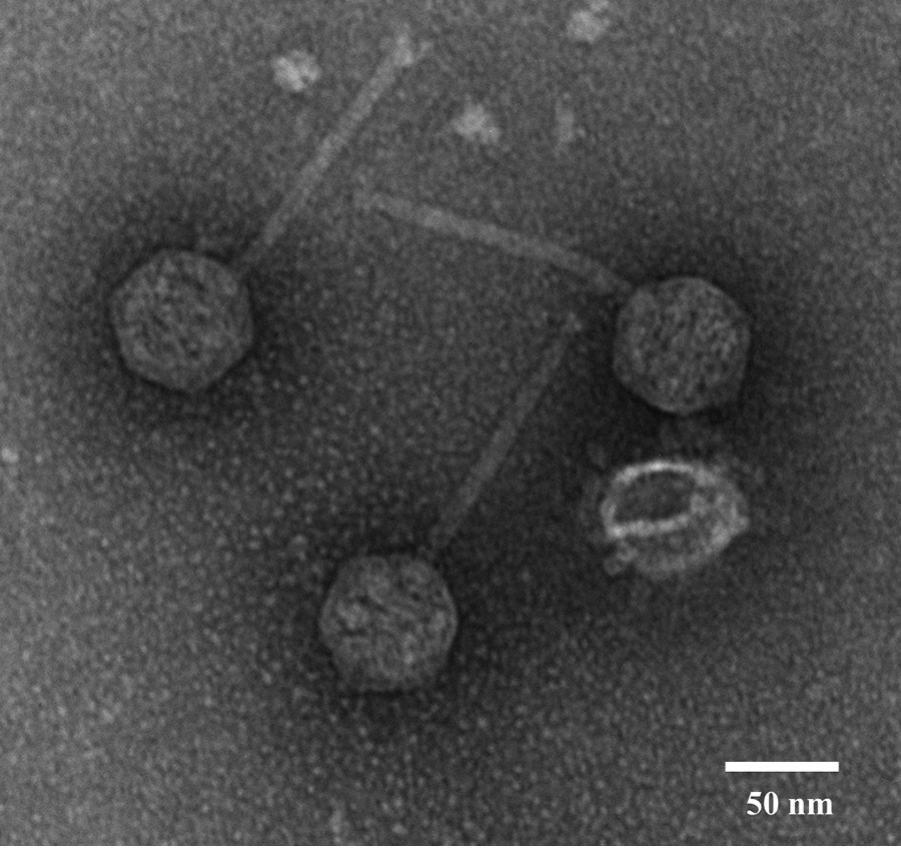
Transmission electron micrograph of the siphovirus Adumb2043. A high-titer lysate (>1.0 × 10^8^ PFU/ml) was negatively stained with 1% uranyl acetate.

Adumb2043 was isolated and purified from a soil sample collected in a public park (under grass) in Colorado Springs, Colorado (38.89966N, 104.69988W), using an enrichment method outlined in the SEA-PHAGES manual (8), with PYCa medium at 30°C and the host A. globiformis B2979-SEA. Under these conditions, Adumb2043 forms uniform circular plaques that are clear during the first 48 h but become turbid with longer incubation times. Transmission electron microscopy analyses reveal Adumb2043 to have an icosahedral head and noncontractile flexible tail (Fig. 1), which are characteristic of the siphoviruses (9).

Genomic DNA extraction was performed at Colorado State University-Pueblo using the Promega Wizard DNA extraction kit, following the manufacturer’s instructions and the SEA-PHAGES manual (8). A sequencing library was prepared at the Pittsburgh Bacteriophage Institute with a NEBNext Ultra II FS kit with dual-indexed barcoding and was sequenced using an Illumina MiSeq platform, which yielded 861,675 single-end 150-base reads. Using Newbler v2.9 with default settings, raw reads were assembled into a single phage contig with shotgun coverage of approximately 2,832-fold; the contig was checked for completeness, accuracy, and phage genomic termini with Consed v29, as described previously (10, 11). The genome has an 11-base 3′ overhang, a G+C content of 67%, and a length of 43,100 bp.

An automated annotation was generated using Glimmer (12) and GeneMark (13), with subsequent manual curation using DNA Master (cobamide2.bio.pitt.edu/computer.htm), Phamerator (14), and Starterator (https://seaphagesbioinformatics.helpdocsonline.com/article-23). Functions for each coding sequence were assigned based on top hits from searches using NCBI BLASTp (15), PhagesDB BLASTp (16), and HHpred (17). Membrane proteins were identified using TMHMM v2.0 (http://www.cbs.dtu.dk/services/TMHMM) and SOSUI (18). All tools were run with default parameters.

Adumb2043 is predicted to have 68 genes; 34 (50%) of these genes have assigned putative functions, whereas 34 (50%) have an unknown (hypothetical) function. In general, the left half of the genome consists of structural genes, whereas the right half contains genes involved in DNA replication. Most (31) of the coding sequences with assigned hypothetical function are found in the right half. Gene 47 codes for an integrase, suggesting that Adumb2043 is a temperate phage, although no repressor has been identified. Of the 68 genes, 67 are predicted to be transcribed in a forward orientation and only 1 (gene 35, with hypothetical function) in a reverse orientation.

A BLASTn search using the nucleotide sequence of the entire Adumb2043 genome to query the phagesdb.org actinophage database returned phages from the AZ cluster as the most similar sequences. Within this cluster, phage Elezi (GenBank accession number MT639653) (92% identity, with 89% query coverage) and phage London (GenBank accession number MT889366) (92% identity, with 89% query coverage) are the closest homologs and phage Maureen (GenBank accession number MH834619.1) (78% identity, with 19% query coverage) is the most distant.

### Data availability.

The complete genome sequence of Adumb2043 has been deposited in GenBank with accession number MT889375, BioProject accession number PRJNA488469, and SRA accession number SRX11564147.
